# Mechanistic analysis of the hardening process of the thorns on stems of *Bougainvillea glabra “Elizabeth Angus”*

**DOI:** 10.3389/fgene.2024.1375488

**Published:** 2024-07-04

**Authors:** Lina Sun, Huaxin Wang, Jinhua Li, Jianying Gong, Shuting Yang, Kaitai Yang, Er Chen, Bing Li, Zhixiang Lu, Qi Chen, Mao Lin

**Affiliations:** ^1^ Guangxi Forestry Research Institute, Nanning, China; ^2^ School of Agriculture and Biology, Shanghai Jiaotong University, Shanghai, China; ^3^ Nanning GoldTech Biotechnology Ltd., Nanning, China

**Keywords:** *Bougainvillea glabra “Elizabeth Angus”*, hardening of the thorns, transcriptome, phenylpropanoid biosynthesis, lignin

## Abstract

**Introduction:** *Bougainvillea glabra* “*Elizabeth Angus*“ is a thorny woody vine or shrub. However, the hard thorns are considered a deficiency in its ornamental value.

**Methods:** To find the genes and pathways related to the hardening process of the thorns on the stems of *B. glabra*, the eukaryotic unreferenced transcriptome sequencing analysis was conducted to explore the 3 stages of the thorn-hardening process. Total RNA was extracted from thorns and stems, and transcriptome libraries were constructed and sequenced using unreferenced Illumina sequencing.

**Results:** Gene function annotation was performed using various databases, resulting in 8937 co-annotated genes. The density distribution of Fragments Per Kilobase of transcript per Million mapped reads (FPKM) depicted the overall gene expression patterns. The study found that stage 2 as the period of highest gene expression activity during the thorns hardening process in *B. glabra*. Differential expression analysis revealed that during thorn-hardening, 1045 genes up-regulated and 391 genes down-regulated significantly in thorns at stage 2 compared to stage 1 (early stage of thorns formation). Meanwhile, 938 genes up-regulated and 784 genes down-regulated significantly in stems. At stage 3, as thorns became harder, 63 genes exhibited notable expression increase and 98 genes’ expression decreased obviously within thorns, and 46 genes up-regulated and 29 genes down-regulated in stems, compared to stage 2. Phenylpropanoid biosynthesis was the key step in the hardening process of the thorns of *B. glabra*. The formation and hardening of thorns on the stem of *B. glabra* was a process in which lignin gradually accumulated in the thorns, and several genes were involved in this process. They include *PAL* (EC:4.3.1.24), *CYP73A* (EC:1.14.14.91), *4CL* (EC:6.2.1.12), *CCR* (EC:1.2.1.44), *CAD* (EC:1.1.1.195) and *POX* (EC:1.11.1.7).

**Discussion:** This transcriptome analysis offers insights into the molecular mechanisms underlying thorns development in this plant species.

## Introduction

*Bougainvillea glabra “Elizabeth Angus”* is an evergreen climbing shrub of Centrospermae (order), Nyctaginaceae (family) (Wu et al., 2003). It is originally from South America, including Brazil, Peru, Argentina’s Chubut Province (Lim, 2014), Bolivia, Ecuador, and Paraguay (Bautista et al., 2022). *Bougainvillea glabra* is widely grown in tropical and subtropical regions in China, where it has been introduced and cultivated for over a century. Presently, in the tropical and subtropical regions of China, such as Guangdong, Hainan, Guangxi, Fujian, and Yunnan Province, *B*. *glabra* has been extensively cultivated demonstrating its adaptability, ease of cultivation, for its colorful flowers (Yanjing, 2010), within the realm of landscaping and ornamental horticulture.

*Bougainvillea glabra* grows as a woody vine or shrub with thorny stems. In the early stage of formation, the thorns on the stems of *B*. *glabra* are green in color and soft in texture. After that, the color gradually deepens and eventually becomes dark brown, and the texture becomes very hard. Ornamental evaluation of *B*. *glabra* involves multiple target traits (Sun Lina et al., 2016), and the rigidity of the thorns on the stems could be considered a target trait. We focus our study on the hardening process of the thorns on the stems of *B*. *glabra* to identify the genes that regulate this process. According to previous studies, it is known that the mechanical strength of plant stems mainly depends on the formation of secondary cell walls. The secondary cell walls are composed mainly of cellulose, hemicellulose, and lignin (Kushwah et al., 2020). Lignin is essential for the mechanical support of plants, for example, lignin deposition is believed to play an important role in cotton fiber development. Another example is that the development of wood properties depends on the lignin deposition (Antonova et al., 2014). Based on these previous studies, it is hypothesized that the synthesis and deposition of cellulose and lignin are closely related to thorn formation in *B*. *glabra*. This research will provide a scientific basis for the cultivation of soft thorns or thorn-free *B*. *glabra* and would provide valuable insights into the developmental regulation of woody modifications in higher plants.

To search for genes and pathways related to the hardening process of *B*. *glabra* thorns, we conducted eukaryotic unreferenced transcriptome sequencing analysis because there is no complete genome information available for this plant species in existing online databases. Our study aims to provide a scientific basis for the cultivation of soft thorns or thorn-free *B. glabra* and to gain insights into the developmental regulation of woody modifications in higher plants.

## Materials and methods

### Plant material

*Bougainvillea glabra* is grown (1 age of these trees) in Nanning, Guangxi Province in China. Three stages in the development of the thorns were identified *viz.*, as given in sample Table 1 below; please refer to the table for a detailed description of the correct stages.

**TABLE 1:** Sampling of thorns and stem from *Bougainvillea glabra* used for RNA isolation in triplicates.

Stage	Thorn morphology/size/days	Sample numbering
Stage 1 thorns	Young soft green/2–4 mm/5 days (Fig1B)	C1–1; C1–2; C1–3
Stage 2 thorns	Young green but hard/5–6 mm/25 days (Fig1C)	C2–1; C2–2; C2–3
Stage 3 thorns	Mature greenish brown hard/7–8 mm/35 days (Fig1D)	C3–1; C3–2; C3–3
Stage 1 stems	Young green stems	J1–1; J1–2; J1–3
Stage 2 stems	Young green stems but hard	J2–1; J2–2; J2–3
Stage 3 stems	Mature hard brown stems	J3–1; J3–2; J3–3

In different stages of thorn formation on the stems, the thorns and stems of *B*. *glabra* were collected for transcriptome analysis. The three stages for analysis include the early stage of thorn formation, the stage in which thorns were hardened, and the stage that is between them. Triplicate samples for each stage were included.

### Transcriptome analysis

#### Library preparation for transcriptome sequencing

A total amount of 1.5 μg RNA per sample was used as an input material for the RNA sample preparations. Sequencing libraries were generated using the NEBNext^®^ Ultra™ RNA Library Prep Kit for Illumina^®^ (NEB, United States), following the manufacturer’s recommendations, and index codes were added to attribute sequences to each sample. In brief, mRNA was purified from total RNA using poly-T oligo-attached magnetic beads. Fragmentation was carried out using divalent cations under elevated temperature in NEBNext First Strand Synthesis Reaction Buffer (5X). First-strand cDNA was synthesized using a random hexamer primer and M-MuLV Reverse Transcriptase (RNase H). Second-strand cDNA synthesis was subsequently performed using DNA polymerase I and RNase H. Remaining overhangs were converted into blunt ends *via* exonuclease/polymerase activities. After adenylation of 3′ ends of DNA fragments, NEBNext Adaptor with a hairpin loop structure was ligated to prepare for hybridization. To select cDNA fragments of preferentially 150–200 bp in length, the library fragments were purified using the AMPure XP system (Beckman Coulter, Beverly, United States). Then, 3 μL USER Enzyme (NEB, United States) was used with size-selected, adaptor-ligated cDNA at 37°C for 15 min, followed by 5 min at 95°C before PCR. Then, PCR was performed with Phusion High-Fidelity DNA polymerase, universal PCR primers, and index (X) primer. At last, PCR products were purified (AMPure XP system), and library quality was assessed on the Agilent Bioanalyzer 2100 system.

### Clustering and sequencing

The clustering of the index-coded samples was performed on a cBot Cluster Generation System using TruSeq PE Cluster Kit v3-cBot-HS (Illumina), according to the manufacturer’s instructions. After cluster generation, the library preparations were sequenced on an Illumina HiSeq platform, and paired-end reads were generated.

### Data analysis

Raw data (raw reads) of fastq format were first processed through in-house perl scripts. In this step, clean data (clean reads) were obtained by removing reads containing the adapter, reads containing ploy-N, and reads whose quality was low from raw data. At the same time, Q20, Q30, and GC content of the clean data were calculated. All the downstream analyses were based on clean data with high quality.

The left ends (read1 files) of fastq format were pooled into one big left.fq file, and the right end (read2 files), into one big right.fq file. The transcriptome assembly was accomplished based on left.fq and right.fq using Trinity (Grabherr et al., 2011) with min kmer cov set to 2 by default and all other parameters set to default. The assembled transcripts were hierarchically clustered to unigenes using shared reads and expressions by Corset (Davidson and Oshlack, 2014).

The correlation of gene expression levels between samples is a crucial indicator to test the reliability of an experiment and to check whether the sample selection is reasonable. It is essential to check the correlation of gene expression levels between samples before performing differential expression analysis. The Pearson correlation coefficient represents the correlation of gene expression levels between samples. The closer the correlation coefficient is to 1, the higher the similarity of expression patterns between samples. A correlation coefficient between 0.8 and 1 indicates a very strong correlation. If the correlation coefficient between samples of biological repeats is lower than 0.8, it means that the samples’ correlation is low. The correlation between pairwise comparisons of the three biological replicates at the same site in the same period is above 0.8, indicating that their respective gene expression levels are similar.

Gene function was annotated based on the NCBI non-redundant protein and nucleotide sequences. Gene function was also annotated by the Pfam (protein family), KOG/COG (clusters of orthologous groups of proteins), Swiss-Prot (a manually annotated and reviewed protein sequence database), KEGG Ortholog database (Kanehisa and Goto, 2000), and Gene Ontology (GO).

### SNP calling

Picard-tools v1.41 and samtools v0.1.18 were used to sort, remove duplicated reads, and merge the bam alignment results of each sample. GATK3 software was used to perform SNP calling. Raw vcf files were filtered using the GATK standard filter method and other parameters (cluster: 3; window size: 35; QD < 2.0 or FS > 60.0 or MQ < 40.0 or SOR >4.0 or MQRankSum < −12.5 or ReadPosRankSum < −8.0 or DP < 10).

### SSR detection and primer design

SSR of the transcriptome was identified using MISA (http://pgrc.ipk-gatersleben.de/misa/misa.html), and the primer for each SSR was designed using Primer3 (http://primer3.sourceforge.net/releases.php). Quantification of gene expression levels was estimated by RSEM (Li and Dewey, 2011) for each sample: 1. clean data were mapped back onto the assembled transcriptome; 2. read count for each gene was obtained from the mapping results.

### Differential expression analysis

Differential expression analysis of two conditions/groups (two biological replicates per condition) was performed using the Differential Expression Sequence (DESeq) R package (1.18.0). DESeq provides statistical routines for determining differential expression in digital gene expression data using a model based on the negative binomial distribution. The resulting *p*-values were adjusted using the Benjamini and Hochberg’s approach for controlling the false discovery rate. Genes with an adjusted *p*-value <0.05 found by DESeq were assigned as differentially expressed.

Prior to differential gene expression analysis, for each sequenced library, the read counts were adjusted using the edgeR program package through one scaling normalized factor. Differential expression analysis of two conditions was performed using the DEGSeq R package (1.20.0). The *p*-values were adjusted using the Benjamini and Hochberg method. A corrected *p*-value of 0.005 and log2 (fold change) of 1 were set as the threshold for significantly differential expression.

### GO and KEGG enrichment analyses of differentially expressed genes

GO enrichment analysis of differentially expressed genes (DEGs) was implemented using the GOseq R package, in which the gene length bias was corrected. GO terms with a corrected *p*-value of less than 0.05 were considered significantly enriched by DEGs. KEGG is a database resource for understanding high-level functions and utilities of the biological system, such as the cell, the organism, and the ecosystem, from molecular-level information, especially large-scale molecular datasets generated by genome sequencing and other high-throughput experimental technologies (http://www.genome.jp/kegg/) (Kanehisa, 2019). We used KOBAS software to test the statistical enrichment of differential expression genes in KEGG pathways.

### PPI analysis of differentially expressed genes

Protein–protein interaction (PPI) analysis of DEGs was based on the STRING database, with known and predicted PPIs. For the species existing in the database, we constructed the networks by extracting the target gene list from the database. Otherwise, Blastx (v2.2.28) was used to align the target gene sequences to the selected reference protein sequences, and then, the networks were built according to the known interaction of selected reference species.

### Transcription factor analysis

Transcription factor (TF) prediction is done using iTAK software, the basic principle of which is to identify TFs by hmmscan using the categorized and defined TF (transcription factor) family and rules in the database.

### RT-qPCR verification

Expression profiling of six genes of the phenylpropanoid pathway was carried out using the reverse transcription fluorogenic quantitative polymerase chain reaction (RT-qPCR). The total RNA from thorns and stems of *B*. *glabra* at three stages was extracted using the HiPure HP Plant RNA Mini Kit (Magen Biotechnology Co., Ltd, R4165-02). cDNA was reverse-transcribed from mRNA using HiScript II Q RT SuperMix for qPCR (+gDNA wiper) (Vazyme Biotech Co., Ltd, R223-01). cDNA and ChamQ Universal SYBR qPCR Master Mix (Vazyme Biotech Co., Ltd, Q711-02) were used for qPCR. Real-time quantitative PCR analysis was conducted using the qTOWER3 system (AnalytikJena, German). The primers used in RT-qPCR are provided in Supplementary Table S1.

### Determination of the lignin content

The samples were dried at 80°C until they reached a constant weight, pulverized, passed through a 40-mesh sieve, and weighed for a certain amount (denoted as W). Acetylation of phenolic hydroxyl groups of lignin in samples was performed. Following the acetylation of phenolic hydroxyl groups within lignin, a discernible absorption peak at 280 nm became evident. Notably, the absorbance measurement at 280 nm exhibited a direct positive correlation with the lignin content present. In this study, the lignin content was characterized by the absorbance value at 280 nm. The formula for converting 280 nm absorbance to lignin content is as follows: Lignin (mg/g) = (ΔA-0.0068)÷0.0347×V×10–3÷W×T = 0.0294×(ΔA-0.0068) ÷0.002 × 50. (V, total volume of the reaction; W, sample quality (dry weight); T, dilution factor; A, measurement of absorbance at 280nm; ΔA, measurement of absorbance at 280 nm of the sample and absorbance value at 280 nm for blank control).

## Results

### Overview of transcriptome analysis

**FIGURE 1:**
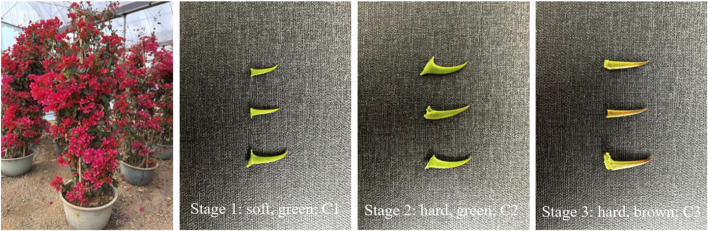
Flowering plants of *B*. *glabra* and the three stages of thorn formation used for the study.

For research purposes, the plants of *B*. *glabra* were selected at three different stages, ranging from thorn formation (stage 1) to thorn hardening (stage 2 to stage 3), as shown in Figure 1. We extracted the total RNA from both thorns and stems and constructed transcriptome libraries for each stage using three biological triplicates of samples. We sequenced the libraries using the Illumina platform and obtained raw reads ranging from 41,266,290 to 49987188 from 18 libraries. The base calling accuracy of more than 99.9% (Q30(%)) for each treatment was over 90%, as shown in Table 2.

**TABLE 2:** List of data output quality.

Sample	Raw reads	Clean reads	Error (%)	Q20 (%)	Q30 (%)	GC content (%)
C1_1	46997486	46652954	0.03	97.38	93.08	43.9
C1_2	49987188	49659918	0.03	97.39	93.05	44.64
C1_3	47513884	4,7221972	0.03	97.38	93.01	44.68
C2_1	47716478	47405948	0.03	97.42	93.11	44.56
C2_2	45417610	45193820	0.03	97.34	92.87	44.71
C2_3	48635900	48360212	0.03	97.35	92.94	44.71
C3_1	45717148	45421208	0.03	97.34	92.95	44.4
C3_2	44137788	43892366	0.03	97.32	92.87	44.4
C3_3	47237234	46963976	0.03	97.37	92.94	44.29
J1_1	43726332	43502176	0.03	97.36	92.95	44.53
J1_2	45084210	44801296	0.03	97.28	92.84	44.65
J1_3	47441554	47145802	0.03	97.49	93.23	44.58
J2_1	46457002	46209204	0.03	97.38	92.98	44.39
J2_2	46184660	45934398	0.03	97.35	92.93	44.46
J2_3	41,266,290	41059518	0.03	97.46	93.19	44.68
J3_1	42274290	42065976	0.03	97.56	93.34	44.31
J3_2	43887862	43646514	0.03	97.56	93.38	44.61
J3_3	46624754	46386220	0.03	97.42	93.13	44.39

Samples of thorns at the three stages: C1, stage 1; C2, stage 2; and C3, stage 3, while stems were at J1, stage 1; J2, stage 2; and J3; stage 3 of maturation.

**FIGURE 2:**
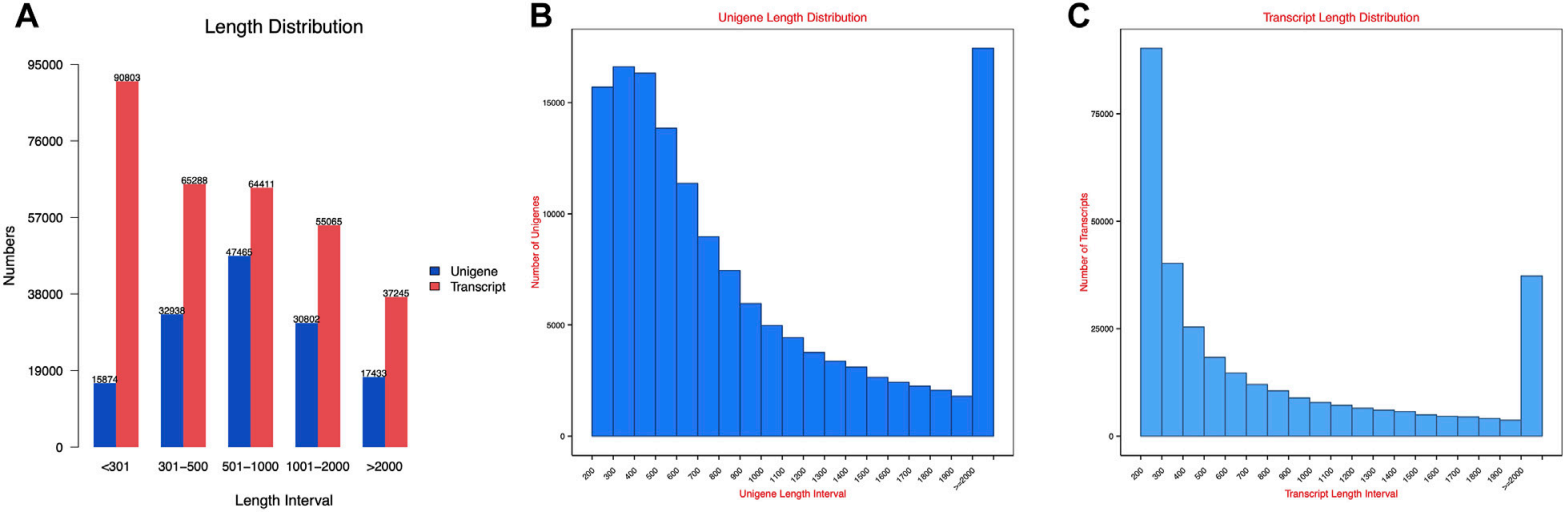
Spliced transcripts and gene sequence length distribution map: **(A)** length distribution; **(B)** unigene length distribution; and **(C)** transcript length distribution.

The transcript sequence assembled by Trinity was used as the reference sequence for subsequent analysis. After hierarchical clustering by Corset, the longest cluster sequence was obtained for subsequent analysis. The lengths of transcripts and clustered sequences were counted separately, and the results are shown in Figure 2.

**FIGURE 3:**
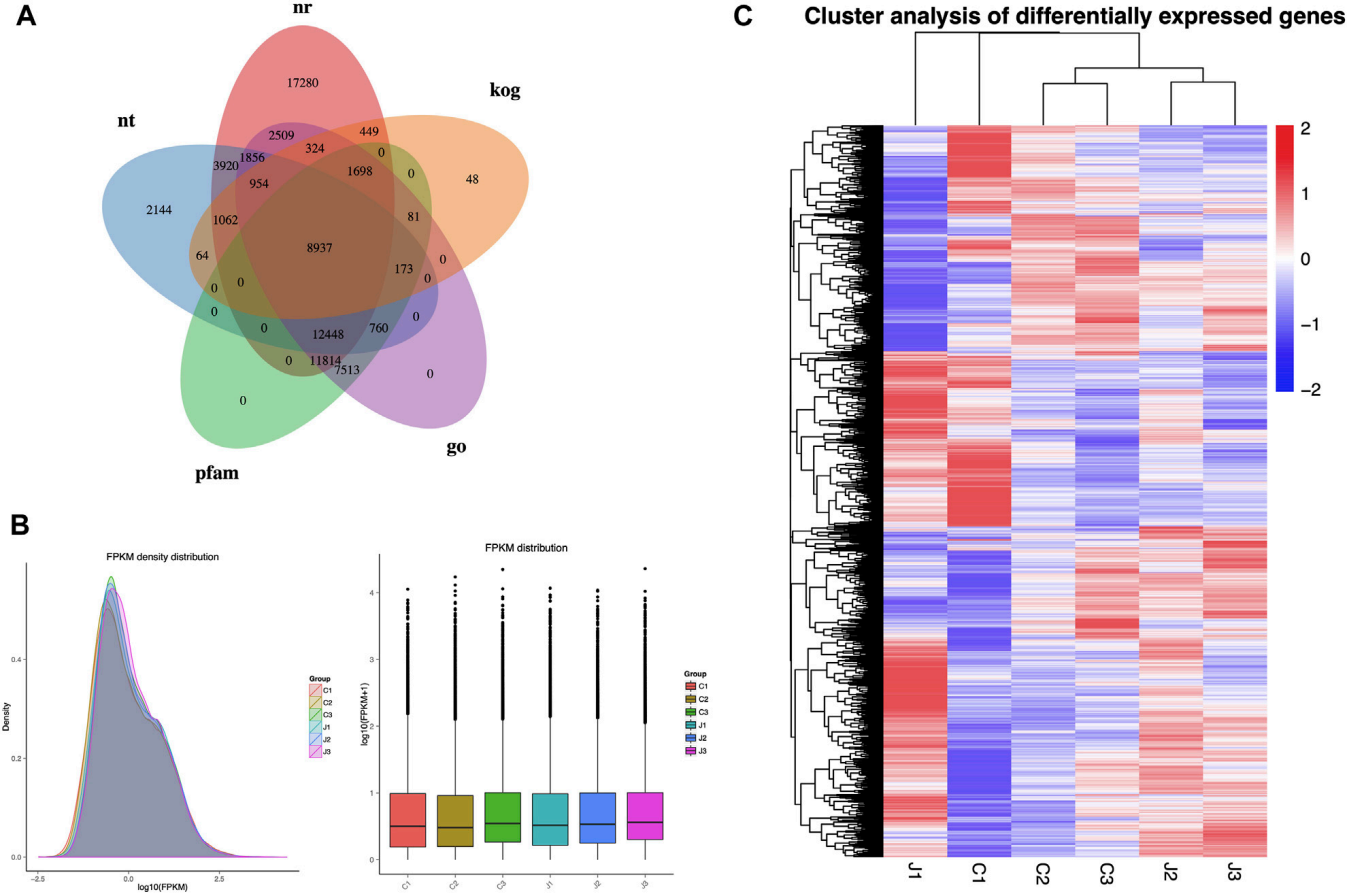
Overview of transcriptome analysis: **(A)** number of genes annotated by each database. **(B)** Comparison chart of gene expression levels in different parts at different periods. **(C)** Cluster analysis of differentially expressed genes. Note: Samples of thorns at the three stages: C1, stage 1; C2, stage 2; and C3, stage 3 while stems were at J1, stage 1; J2, stage 2, and J3, stage 3.

Gene function was annotated using various databases including NCBI, Pfam, KOG/COG, Swiss-Prot, KEGG Ortholog, and GO. A total of 8,937 genes were co-annotated using these databases (as shown in Figure 3A). The FPKM density distribution reflects the gene expression pattern of each sample as a whole. The graph shows a non-standard normal distribution with an area of 1, indicating that the sum of probabilities is 1. The peak of the density distribution curve represents the highest number of genes at the expression level (Figure 3B). In this experiment, hierarchical clustering analysis was performed using the FPKM values of differential genes under different experimental conditions as expression levels. Genes in the same cluster exhibit similar expression level changes under various treatment conditions. Genes within the same group have similar expression patterns and might participate in the same biological processes or have similar functions. As shown in Figure 3C, the gene expression patterns in stages 2 and 3 are significantly different from those in stage 1, both in thorns and stems. Furthermore, the gene expression patterns are notably different between thorns and stems.

### Differentially expressed gene analysis

**FIGURE 4:**
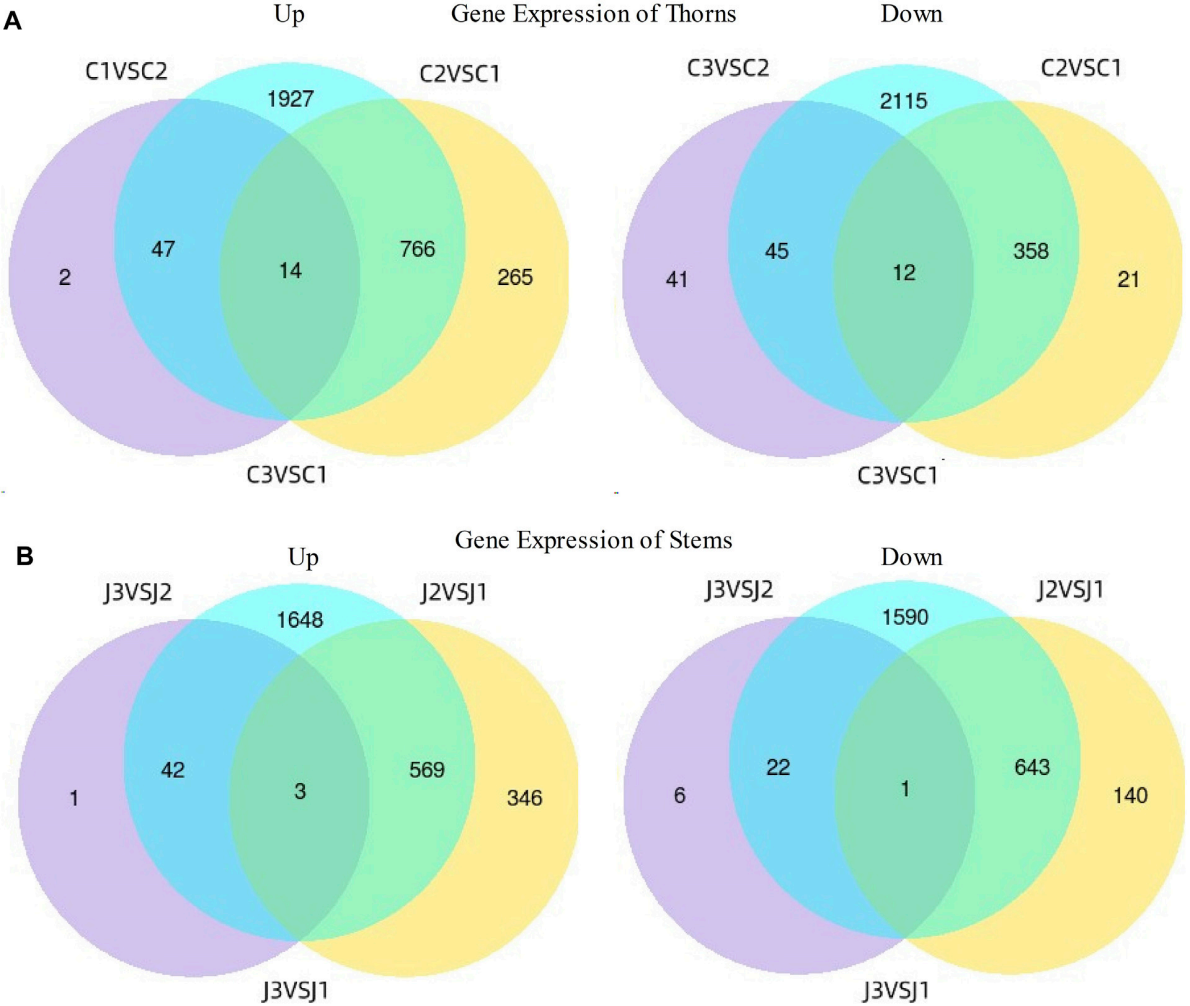
Differences in gene expression at three stages. **(A)** Gene expression of thorns. **(B)** Gene expression of stems. Note: Samples of thorns at the three stages- C1- stage 1; C2- stage 2 and C3- stage 3 while stems were at J1- stage 1; J2- stage 2 and J3- stage 3.

Compared to the thorn formation stage (stage 1), at the stage of thorns turning hard (stage 2), a total of 1,045 genes were upregulated, and 391 genes were downregulated significantly in thorns (Figure 4A), while 918 genes were upregulated and 784 genes were downregulated markedly in stems (Figure 4B). As the thorns become hard and turn brown (stage 3), a total of 63 genes exhibited a noteworthy increase in expression and 98 genes showed a significant decrease in expression in the thorns (Figure 4A), while 46 genes showed a significant upregulation in expression and 29 genes showed downregulation within the stems, compared with the stage 2 (Figure 4B). The results indicate that stage 2 is the most active period of gene expression during the hardening process of *B*. *glabra* thorns.

### GO enrichment analysis

**FIGURE 5:**
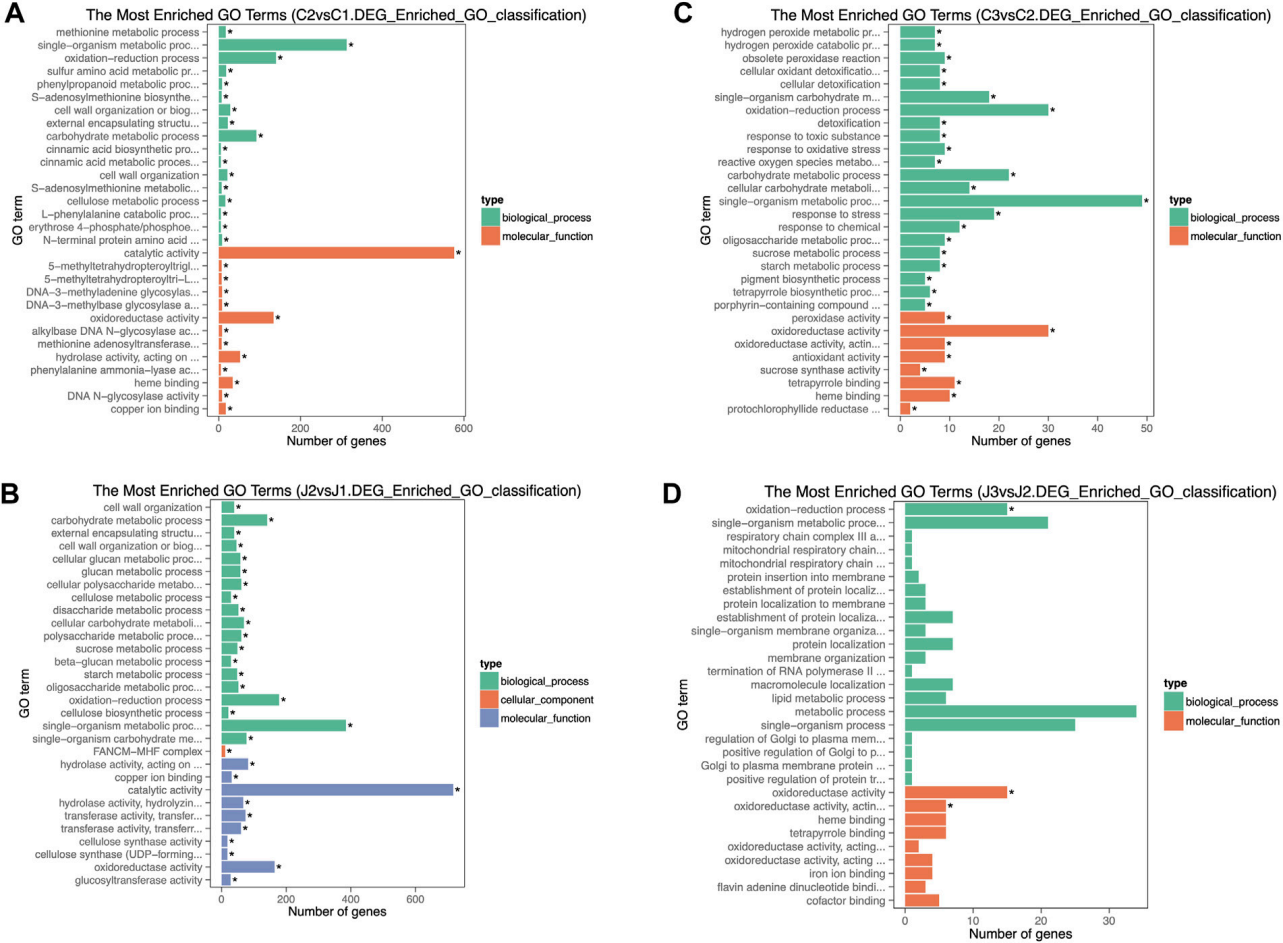
GO gene enrichment analysis shown for stages of thorn and stem maturation: **(A)** C2 vs. C1. **(B)** J2 vs. J1. **(C)** C3 vs. C2. **(D)** J3 vs. J2.

Active gene expression means vigorous biosynthesis and metabolism in the organism. According to GO enrichment analysis, at the stage of thorns turning hard, the three most gene-enriched biological processes in thorns (C2) were single-organism metabolism, carbohydrate metabolism, and the oxidation–reduction process, compared with the early stage of thorn formation (C1). At the same time, the molecular function exhibiting the highest degree of gene enrichment was catalytic activity (Figure 5A). The gene enrichment in stem samples was generally consistent with that observed in thorn samples (Figure 5B). Comparing the gene enrichment analysis at stage 3 with stage 2 of thorns (C3 VS C2), the bioprocess of metabolism was the most enriched, while the molecular function of oxidoreductase activity was enhanced as the top activity. The number of genes enriched in these biological processes decreased significantly when the thorns entered 2–3 stage. However, the biological processes in which genes are mainly enriched are still the same three processes—the single organism metabolism process, carbohydrate metabolism process, and oxidation–reduction process (Figure 5C). The situation of gene enrichment in the thorn (J3 VS J2) samples was comparable to what was noted in the stem samples (Figure 5D).

**FIGURE 6:**
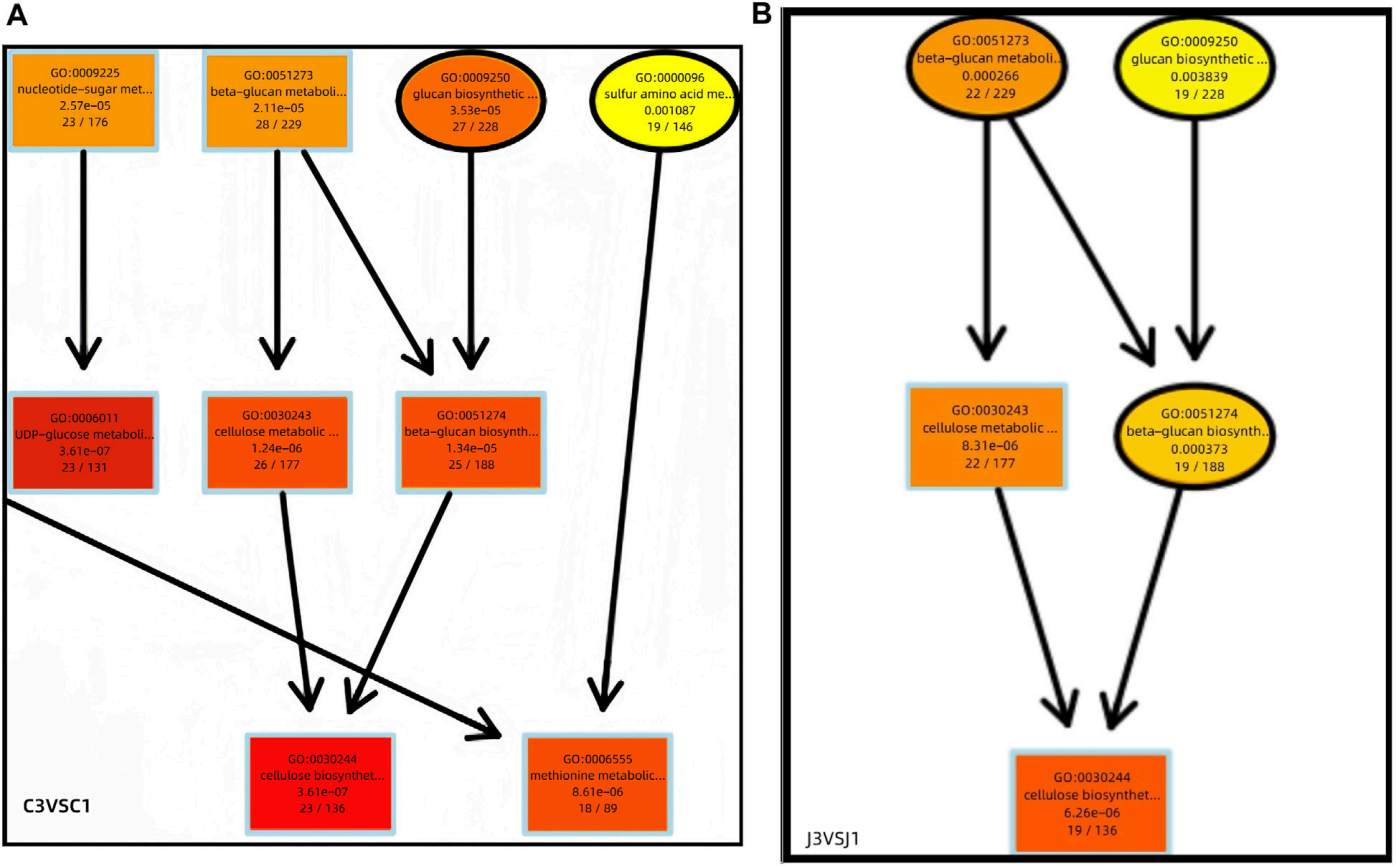
Differential gene GO enrichment DAG plot (partial view). **(A)** C3 VS C1. **(B)** J3 VS J1.

A directed acyclic graph (DAG) was drawn based on the results of differential gene GO enrichment analysis. Further comprehensive analysis of the biological process from stage 1 to stage 3 shows that the synthesis of cellulose is significantly enriched in this process (Figure 6). It is found that genes related to cellulose synthesis are significantly enriched in thorns during this process, whereas genes related to cellulose degradation and synthesis are significantly enriched in the stems. We speculate that degraded cellulose in the stem may be transported to the thorns and participate in their hardening process.

### KEGG enrichment and RT-qPCR analysis and determination of lignin

**FIGURE 7:**
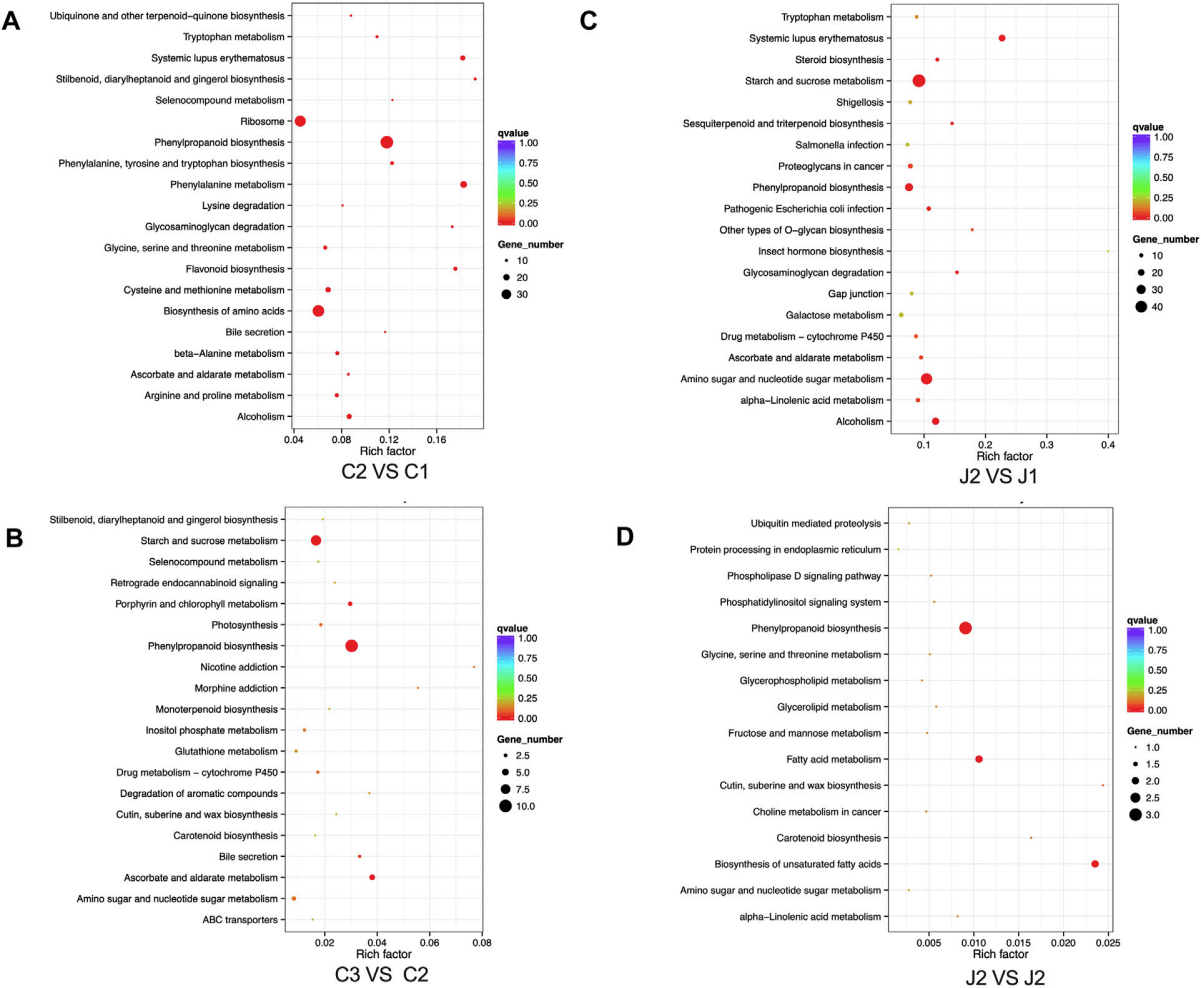
KEGG gene enrichment analysis in thorns and stems: **(A)** KEGG gene enrichment analysis in thorns from stage 1 to stage 2. **(B)** KEGG gene enrichment analysis in thorns from stage 2 to stage 3. **(C)** KEGG gene enrichment analysis in stems from stage 1 to stage 2. **(D)** KEGG gene enrichment analysis in stems from stage 2 to stage 3.

**FIGURE 8:**
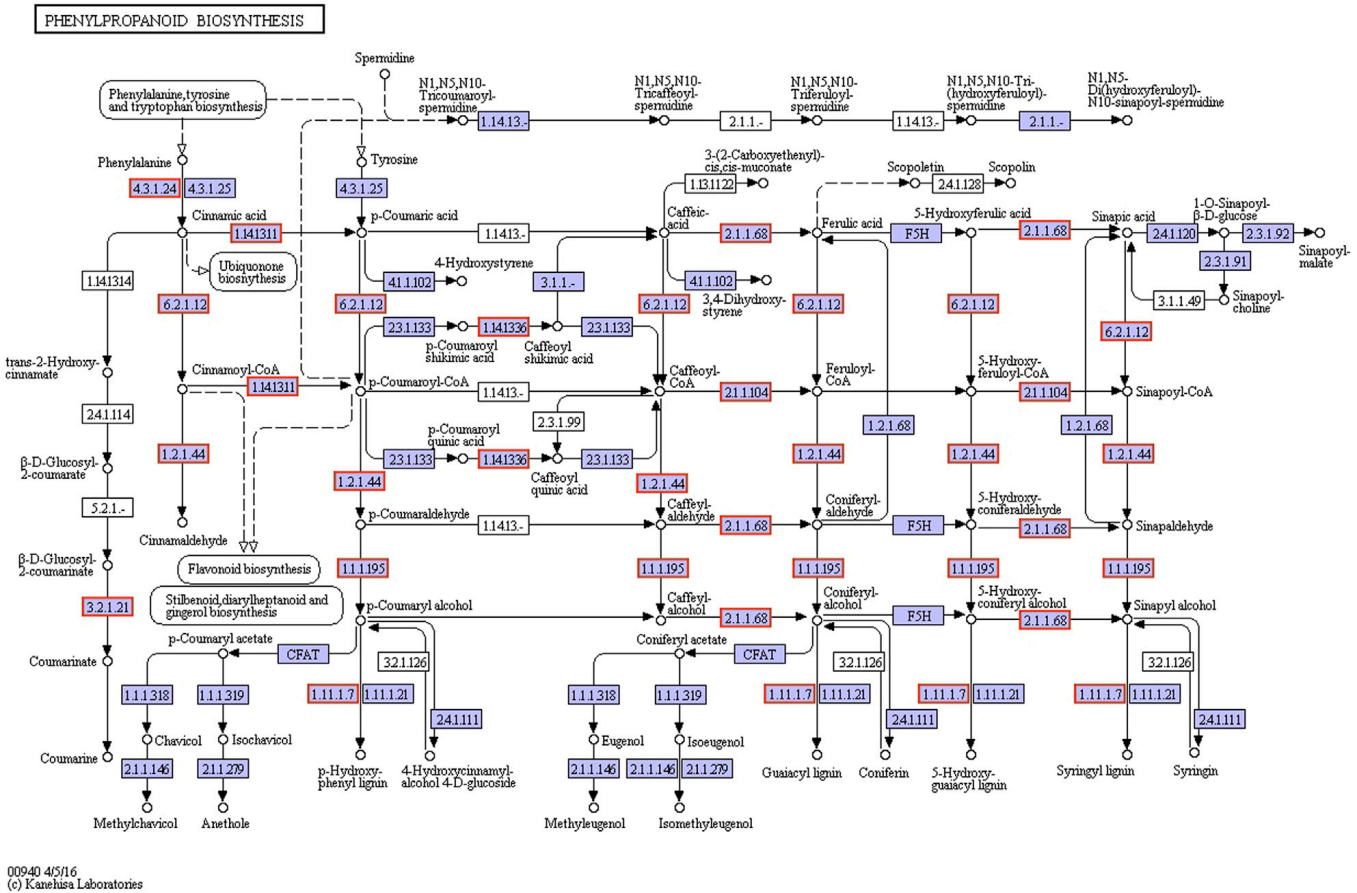
Upregulated genes in phenylpropanoid biosynthesis (C2 VS C1) (Ref: 231673).

**FIGURE 9:**
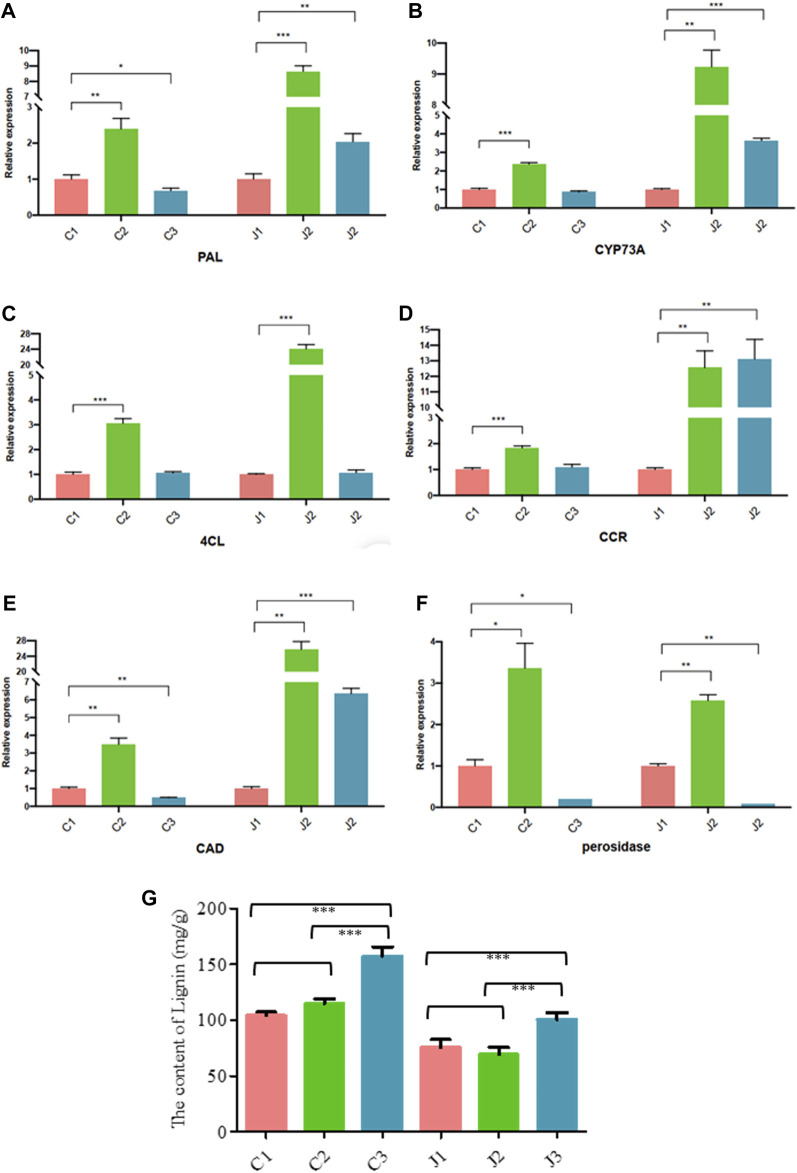
Expression profiles of genes using RT-qPCR in the thorns [C1, C2, and C3] and stems [J1, J2, and J3] at three stages of maturation. **(A)** The genes assayed were *PAL* **(B)**, *CYP37A* **(C)**, *4CL* **(D)**, *CCR* **(E)**, *CAD* **(F)**, and *POX*. **(G)** Lignin content in the thorns [C1, C2, and C3] and stems [J1, J2, and J3] at three stages of maturation.

Through KEGG enrichment analysis, we found that phenylpropanoid biosynthesis was a key step in the hardening process of the thorns of *B*. *glabra*. From stage 1 to stage 3, in thorns, the most significantly enriched pathway was the phenylpropanoid biosynthesis (Figure 7; Supplementary Tables S2–5). From stage 1 to stage 2, many genes in phenylpropanoid biosynthesis were upregulated remarkably in thorns (Figure 8). The above results were also verified by RT-qPCR of the important enzymes of the pathway. It indicates that *PAL* (EC:4.3.1.24, K10775, phenylalanine ammonia-lyase) (Figure 9A), *CYP73A* (EC:1.14.14.91, K00487, trans-cinnamate 4-monooxygenase) (Figure 9B), *4CL* (EC:6.2.1.12, K01904, 4-coumarate-CoA ligase) (Figure 9C), *CCR* (EC:1.2.1.44, K09753, cinnamoyl-CoA reductase) (Figure 9D), *CAD* (EC:1.1.1.195, K00083, cinnamyl-alcohol dehydrogenase) (Figure 9E), and *POX* (EC:1.11.1.7, K00430) (Figure 9F) were the key genes for the phenylpropanoid biosynthesis pathway, leading to the structural component of plant secondary cell walls and lignin biosynthesis. The expression levels of these genes exhibited a notable upregulation during the second stage compared with the first stage. The phenylpropanoid biosynthesis is the key step of lignin biosynthesis. The determination of the lignin content in thorns and stems at three stages (Figure 9G) also agreed with the results of transcriptome analysis and RT-qPCR. To sum up, phenylpropanoid biosynthesis is a key step in the hardening process of the thorns of *B*. *glabra* as it was upregulated during maturation with maximum gene expression of the key enzymes during stage 2 of maturation.

### Transcription factors in transcriptomes and correlation analysis with lignin biosynthesis

**FIGURE 10:**
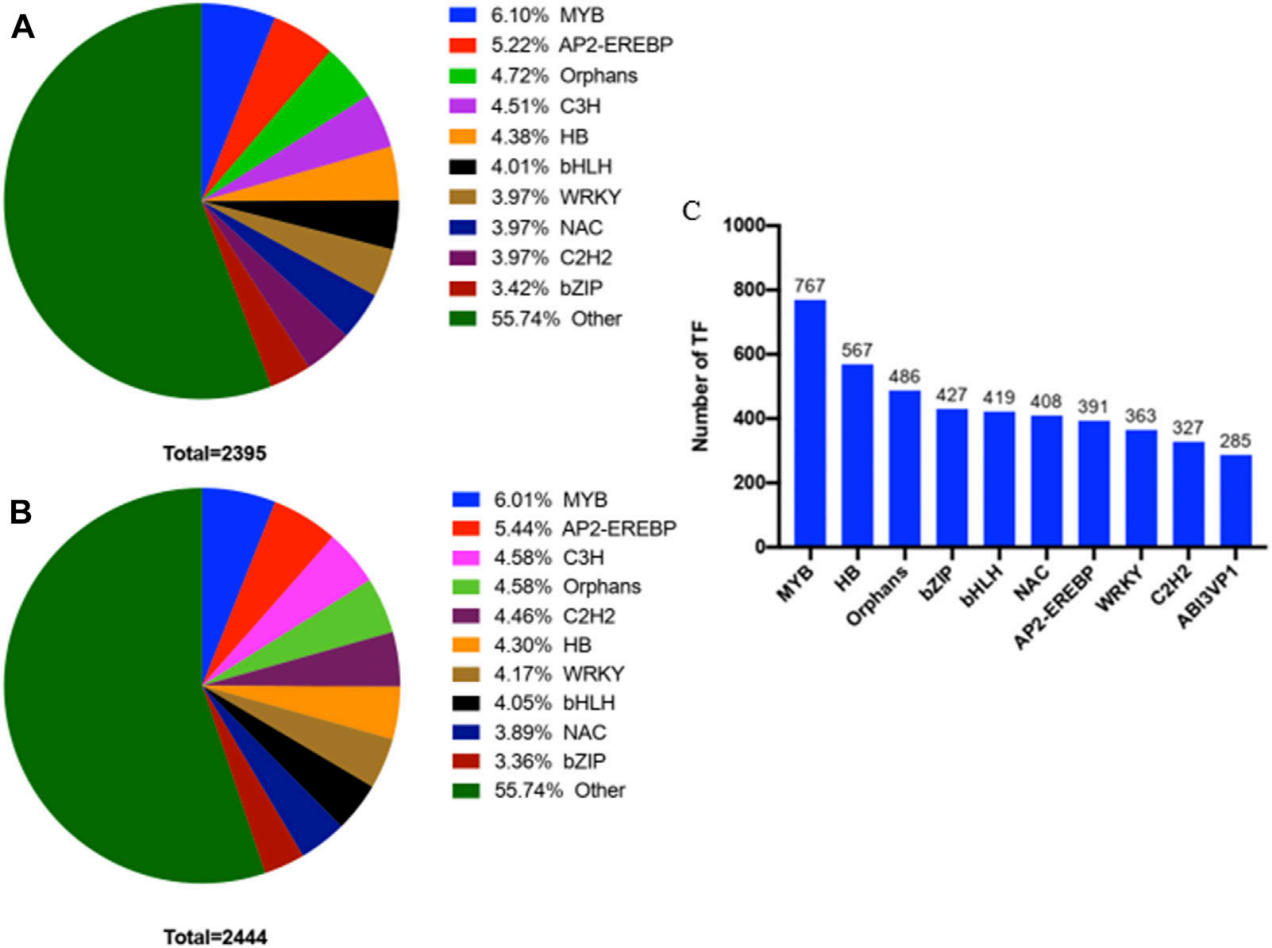
Statistical map of transcription factors with significant differences in different tissues **(A)** Transcription factors with significant differential expression in thorns from the first stage to the third stage. **(B)** Transcription factors with significant differential expression in stems from the first stage to the third stage. **(C)** The top 10 TFs correlated with phenylpropanoid synthesis.

From the first stage to the third stage of thorn formation, 2,395 TFs were completely identified in thorns. They included MYB, AP2-EREBP, orphans, C3H, HB, bHLH, WRKY, NAC, C2H2, and bZIP (Figure 10A). In stems, 2,444 TFs were completely identified. They included similar types of TFs as thorns, differing only in the percentage of TFs in each class from thorns (Figure 10B). From the first stage to the second stage of the thorn formation, 39 genes were significantly altered in the phenylpropanoid biosynthesis pathway in thorns (Supplementary Table S2). By correlation analysis, there were many TFs with correlation coefficients greater than 0.8 with these genes. These TFs MYB and HB are the most numerous (Figure 10C).

## Discussion

In the process of thorns’ hardening, the synthesis of cellulose is significantly enriched by GO analysis, and the phenylpropanoid biosynthesis is enriched by KEGG analysis (Figure 8). Both the synthesis of cellulose (Figure 6) and lignin (Figure 9) are highly related to the secondary cell wall development, which is supposed to be the important process of thorns’ hardening.

From stage 1 to stage 3, a continuous accumulation of lignin in thorns of *B*. *glabra* was observed.

Lignin constitutes a polymer resulting from the intricate polymerization of distinct lignin monomers. The process of lignin biosynthesis is orchestrated through the integration of the phenylalanine metabolic pathway and the dedicated lignin-specific pathway. The synthesis of lignin from phenylalanine can be divided into four steps. First, phenylalanine forms p-coumaric acid under the catalysis of phenylalanine ammonia-lyase (*PAL*) and cinnamic acid 4-hydroxylase (*C4H*). Next, p-coumaric acid forms caffeic acid under the catalysis of p-coumarate 3-hydroxylase (*C3H*), caffeic acid forms ferulic acid under the catalysis of caffeic acid O-methyltransferase (*COMT*), and ferulic acid forms 5-hydroxy-ferulic acid under the catalysis of ferulate-5-hydroxylase (*F5H*) and then forms sinapic acid by the catalysis of caffeic acid O-methyltransferase. Subsequently, the phenolic acids previously elucidated undergo catalytic transformation facilitated by a sequence of enzymes. Commencing with 4-coumarate CoA ligase (*4CL*), the process advances through cinnamoyl-CoA reductase (*CCR*) and culminates in cinnamyl alcohol dehydrogenase/sinapyl alcohol dehydrogenase (*CAD*/*SAD*)-mediated reactions. These orchestrated enzymatic steps culminate in the synthesis of distinctive compounds, namely, p-coumaryl alcohol, caffeoyl alcohol, coniferyl alcohol, 5-hydroxy-coniferyl alcohol, and sinapyl alcohol. Ultimately, the trimeric units of lignin—namely, p-coumaryl alcohol, coniferyl alcohol, and sinapyl alcohol—underwent a process of polymerization catalyzed by peroxidase (*POD*/*PER*/*PRX*) or laccase (*LAC*). This orchestrated transformation yielded three distinct high-molecular weight lignin polymers: p-hydroxyphenyl lignin, guaiacyl lignin, and syringyl lignin (Yoon et al., 2015). From the formation to hardening of thorns on the stems of *B*. *glabra*, the genes encoded *PAL*, *CCR*, *4CL*, *CAD*, and *POX* are upregulated during this process. At the same time, lignin gradually accumulated in thorns and stems. It was confirmed that the hardening process of the thorns on stems of *B*. *glabra* depended on the lignin accumulation. The lignin accumulation in the thorns of *B*. *glabra* relied on the phenylpropanoid biosynthesis pathway.

TFs play a crucial role in the regulation of gene expression. By binding to specific DNA sequences in the promoter regions of target genes, TFs activate or repress the transcription of those genes to achieve the control of genes expression in a coordinated and regulated manner (Yusuf et al., 2012). TFs are essential for the normal development and functioning of plants. For example, a class of TFs, NACs, which play important roles in regulating plant growth and development, are involved in secondary cell wall development, seed germination, root development, leaf senescence, floral organ formation, and fruit ripening, as well as mediating plant responses to low temperature, high temperature, drought, flooding, high salt, and disease (Han et al., 2023).

When it comes to the regulation of lignin synthesis, current research mainly focuses on two types of TFs, NAC and MYB (Figure 10). In *Arabidopsis thaliana*, a three-level regulatory network composed of NAC and MYB jointly regulates the synthesis of lignin, cellulose, and hemicellulose during secondary cell wall thickening in *A*. *thaliana* (Nakano et al., 2015). In addition to NAC and MYB TFs, lignin synthesis was also regulated by WRKY, bHLH, LBD, LIM, and microRNA, etc. (Behr et al., 2019). Based on the transcriptome analysis of the three stages, we can further screen the regulatory factors that regulate the accumulation of lignin in thorns, to provide a more scientific basis for the cultivation of soft thorns or thorn-free *B*. *glabra*. In the other study, the content of soluble sugar and starch was measured during the growth and development of the thorn, and transcriptome sequencing of the thorn segment, non-thorn segment, apex, and root tip was performed at five distinct stages of thorn formation. The findings indicate that as *Gleditsia sinensis* underwent maturation, there was a discernible pattern in the soluble sugar content across various plant components, including roots, hypocotyls, thorn stems, thornless stems, and leaves. Additionally, the starch content in roots and leaves exhibited an initial increase, followed by a subsequent decline upon the establishment of the fundamental thorn structure. Notably, the overall trajectory of both soluble sugar and starch content in *G*. *sinensis* demonstrated a consistent decrease, amounting to a reduction of 59.26% and 84.56%, respectively. Genes such as MYB-like, YABBY2, growth-regulating factor 3 (GRF3), TCP2, zinc transporter 8, and additional 25 genes are posited to be associated with the regulation and promotion of thorn maintenance and growth. The GO enrichment analysis conducted on DEGs between stems exhibiting thorns and those without revealed a notable enrichment of DEGs associated with biological processes indicative of positive regulation of development, specifically in the context of heterochronic events (GO:0045962) and the positive modulation of photomorphogenesis (GO:2000306), among other relevant biological process terms. This observation suggests a correlation between the regulation of developmental initiation in *G*. *sinensis* and the involvement of TCP TFs (Xiao et al., 2023). In our study, we focused on the lignin accumulation relied on the phenylpropanoid biosynthesis pathway. It is believed that lignin accumulation was the key step to forming the thorns. Until now, we cannot find any varieties of *B*. *glabra* that have no thorns. The above studies give us some insights for future studies where we can focus on TFs that act on the phenylalanine metabolic pathway. Lignin accumulation was reduced in thorns using RNA interference and other means, with a view to breeding varieties with soft thorns. Furthermore, if the lignin transport from stems to thorns could be blocked, it might be possible to produce thornless varieties of *B*. *glabra*.

## Conclusion

The process of thorn formation and hardening on the stem of *B*. *glabra* involves the gradual accumulation of lignin in the thorns. Several genes are involved in this process, including *PAL* (phenylalanine ammonia-lyase), *CYP73A* (trans-cinnamate 4-monooxygenase), *4CL* (reductase4-coumarate-CoA ligase), *CCR* (cinnamoyl-CoA reductase), *CAD* (cinnamyl-alcohol dehydrogenase), and *POX* (peroxidase). These genes play a crucial role in lignin synthesis and accumulation, which occurs through the phenylpropanoid biosynthesis pathways.

## Data Availability

The datasets presented in this study can be found in online repositories. The names of the repository/repositories and accession number(s) can be found at: https://www.ncbi.nlm.nih.gov/genbank/, PRJNA1015025.

## Supplementary material

SUPPLEMENTARY TABLE S1 Primers used in RT-qPCR.

SUPPLEMENTARY TABLE S2 KEGG-enriched pathways: top 10 (C2 VS C1).

SUPPLEMENTARY TABLE S3 KEGG-enriched pathways: top 10 (C3 VS C2).

SUPPLEMENTARY TABLE S4 KEGG-enriched pathways: top 10 (J2 VS J1).

SUPPLEMENTARY TABLE S5 KEGG-enriched pathways: top 10 (J3 VS J2).
