# Supplementary material for: Mechanistic analysis of the hardening process of the thorns on stems of Bougainvillea glabra “Elizabeth Angus”
Source: Front Genet. 2024 Jul 4;15:1375488. doi: 10.3389/fgene.2024.1375488 (PMC11254801; doi:10.3389/fgene.2024.1375488)
Supplement: Supplementary file 1 [file Table1.pdf]

Supplementary Table

Supplementary Table 1 The primers used in RT-qPCR

Gene name	Primers name	Sequence(5'to3')
actin	actin F	TAGACCCTCCTATCCAAACA
	actin R	TTTTCCAGCCTTCACTTATC
PAL	c59052_g5_i1 F	CTTGAGCCACCGTGAGAGTT
	c59052_g5_i1 R	ACCACCACCAGCACCAGAA
CYP73A	c49534_g1_i1 F	CCAGATAACAGAGCCAGACACA
	c49534_g1_i1 R	CCACCAGGCATTCACCAGAA
4CL	c77978_g1_i0 2F	GTGGCACAACAAGTGGATGG
	c77978_g1_i0 2R	GCGTAGAGCACACAGCAGTA
CCR	c48436_g0_i0 F	ACAACCAAGCCACCACTACC
	c48436_g0_i0 R	ACCATCCATGAAGCAATGAACC
CAD	c35663_g1_i0 F	TTGGTGTGATCGTTGGATGTTG
	c35663_g1_i0 R	CGCTGCTTGTTCTGATGCC
peroxiddase	c53159_g0_i0 F	AAGCAACCAGGCAGAGAAGG
	c53159_g0_i0 R	ATCAGCACAAGAGACGATTCCT

pathway_term	rich_factor	qvalue	gene_number
Phenylpropanoid biosynthesis	0.118182	1.03E-12	39
Phenylalanine metabolism	0.182609	8.35E-10	21
Systemic lupus erythematosus	0.181818	1.82E-07	16
Flavonoid biosynthesis	0.175676	6.07E-06	13
Biosynthesis of amino acids	0.060606	4.05E-05	36
Stilbenoid, diarylheptanoid and gingerol biosynthesis	0.192308	5.78E-05	10
Phenylalanine, tyrosine and tryptophan biosynthesis	0.122449	0.000308	12
Glycosaminoglycan degradation	0.173077	0.000308	9
Alcoholism	0.086486	0.000522	16
Tryptophan metabolism	0.10989	0.002752	10

Supplementary Table 2 KEGG enriched pathways Top 10 (C2 VS C1)

Supplementary Table 3 KEGG enriched pathways Top 10 (C3 VS C2)

pathway_term	rich_factor	qvalue	gene_number
Phenylpropanoid biosynthesis	0.03030303	1.03E-08	10
Starch and sucrose metabolism	0.016736402	2.92E-05	8
Ascorbate and aldarate metabolism	0.038095238	0.000449382	4
Porphyrin and chlorophyll metabolism	0.02970297	0.00703741	3
Bile secretion	0.033333333	0.038404153	2
Photosynthesis	0.018518519	0.092852753	2
Drug metabolism - cytochrome P450	0.017391304	0.092852753	2
Nicotine addiction	0.076923077	0.103114846	1
Amino sugar and nucleotide sugar metabolism	0.008241758	0.103114846	3
Inositol phosphate metabolism	0.012269939	0.112836371	2

Supplementary Table 4 KEGG enriched pathways Top 10 (J2 VS J1)

pathway_term	rich_factor	qvalue	gene_number
Systemic lupus erythematosus	0.227273	1.25E-08	20
Amino sugar and nucleotide sugar metabolism	0.104396	4.39E-08	38
Starch and sucrose metabolism	0.09205	4.80E-08	44
Alcoholism	0.118919	1.48E-05	22
Phenylpropanoid biosynthesis	0.075758	0.003037	25
Glycosaminoglycan degradation	0.153846	0.015022	8
Pathogenic Escherichia coli infection	0.107843	0.018301	11
Steroid biosynthesis	0.121622	0.023345	9
Sesquiterpenoid and triterpenoid biosynthesis	0.145833	0.030822	7
alpha-Linolenic acid metabolism	0.090164	0.048679	11

Supplementary Table 5 KEGG enriched pathways Top 10 (J3 VS J2)

pathway_term	rich_factor	qvalue	gene_number
Phenylpropanoid biosynthesis	0.009090909	0.006417295	3
Biosynthesis of unsaturated fatty acids	0.023529412	0.006417295	2
Fatty acid metabolism	0.010582011	0.020138479	2
Cutin, suberine and wax biosynthesis	0.024390244	0.080045911	1
Carotenoid biosynthesis	0.016393443	0.094100738	1
alpha-Linolenic acid metabolism	0.008196721	0.130751263	1
Glycerolipid metabolism	0.005813953	0.130751263	1
Phosphatidylinositol signaling system	0.005586592	0.130751263	1
Phospholipase D signaling pathway	0.005235602	0.130751263	1
Glycine, serine and threonine metabolism	0.005102041	0.130751263	1
